# Sequencing and characterization of the transcriptome of half-smooth tongue sole (*Cynoglossus semilaevis*)

**DOI:** 10.1186/1471-2164-15-470

**Published:** 2014-06-13

**Authors:** Wenji Wang, Qilin Yi, Liman Ma, Xiaosu Zhou, Haitao Zhao, Xubo Wang, Jie Qi, Haiyang Yu, Zhigang Wang, Quanqi Zhang

**Affiliations:** College of Marine Life Sciences, Ocean University of China, Key Laboratory of Marine Genetics and Breeding, Ministry of Education, Qingdao, 266003 China

**Keywords:** Cynoglossus semilaevis, Half-smooth tongue sole, Transcriptome, SNP, SSR

## Abstract

**Background:**

Half-smooth tongue sole (*Cynoglossus semilaevis*) is a valuable fish for aquaculture in China. This fish exhibits sexual dimorphism, particularly different growth rates and body sizes between two genders. Thus, *C. semilaevis* is a good model that can be used to investigate mechanisms responsible for such dimorphism, this model can also be utilized to answer fundamental questions in evolution and applied fields of aquaculture. Hence, advances in second-generation sequencing technology, such as 454 pyrosequencing, could provide a robust tool to study the genome characteristics of non-model species.

**Results:**

In this study, *C. semilaevis* was subjected to *de novo* transcriptome sequencing and characterization. A total of 749,954 reads were generated using a single 454 sequencing run in a full PicoTiter plate. These reads were then assembled into 62,632 contigs with a 10-fold average sequencing coverage. A total of 26,589 sequences were successfully annotated based on sequence similarities; among these sequences, 3,451 transcripts exhibited gene ontology terms and 2,362 showed enzyme commissions associated with 186 pathways from Kyoto Encyclopedia of Gene and Genomes pathways. A search of repetitive elements was performed, and 1,898 transposable elements were identified. Approximately 7,800 simple-sequence repeats and 21,234 single-nucleotide polymorphisms were also detected.

**Conclusions:**

Our data provided an integrated and comprehensive transcriptome resource for *C. semilaevis*. These data could be used for further research in population genetics, gene function, and tissue-specific gene expressions.

**Electronic supplementary material:**

The online version of this article (doi: 10.1186/1471-2164-15-470) contains supplementary material, which is available to authorized users.

## Background

Half-smooth tongue sole (*Cynoglossus semilaevis*) is a large flatfish species naturally distributes in East Asia [1]. This species has been considered as one of the most flavored species, which shows special advantages in a mild taste. Owing to limited wild resources, this flatfish has become an important farmed fish in China [2]. Studies on this valuable aquatic fish have focused on the development of genetic markers, construction of genetic maps, and characterization of functional genes involved in growth, reproduction, stress, and immunity [3–6]. Recently, whole genome sequencing of *C. semilaevis* has been completed which made the study of species into a new phase [7].

Advances in high-throughput sequencing technologies have facilitated the studies on the genome and transcriptome of a non-model organism, such as *C. semilaevis*. Massively parallel 454 pyrosequencing, which shows its long-read characteristics (>400 bp), can be performed for *de novo* transcriptome analysis [8]. Using 454 pyrosequencing, researchers sequenced and characterized the transcriptomes of many species [9–12].

The present study aimed to characterize the transcriptome of *C. semilaevis*. A multi-tissue and multi-individual library was constructed and sequenced using a 454 GS FLX titanium platform. Sequence assembly, gene annotation, transposable element (TE) analysis, and marker identification were performed.

## Results and discussion

### Sequence analysis and assembly

**Figure 1:**
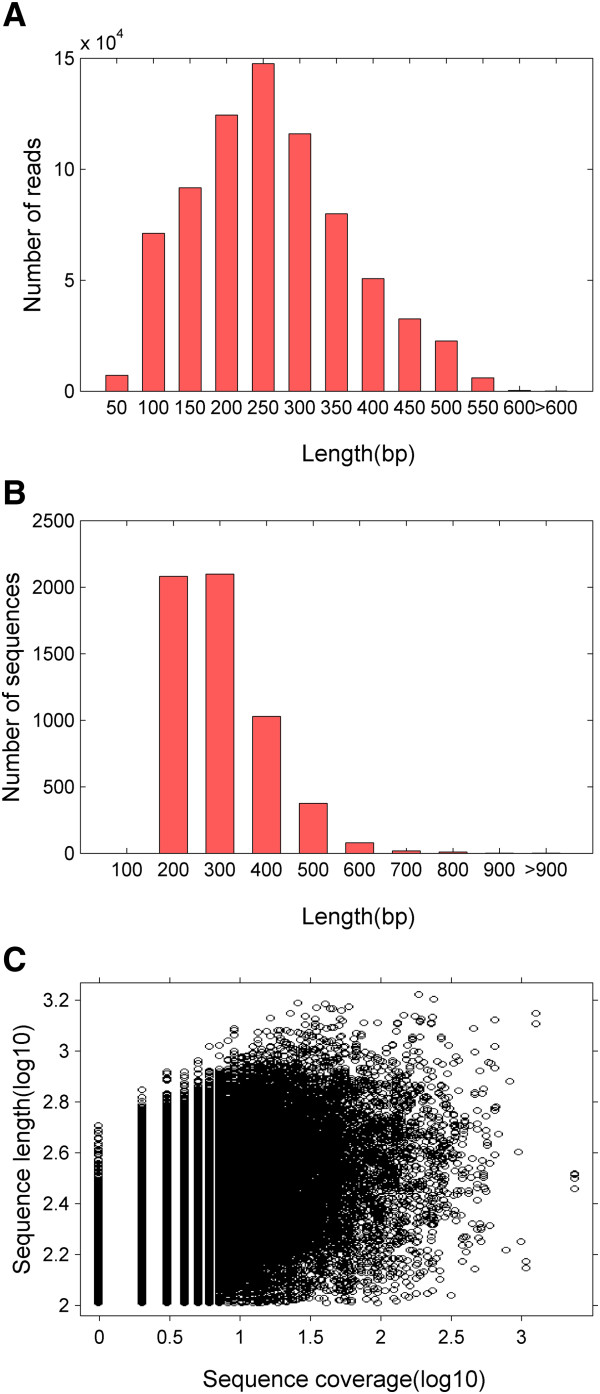
**Overview of the** ***C. semilaevis*** **transcriptome sequencing and assembly. (A)** Size distribution for raw reads. **(B)** Size distribution for contigs. **(C)** Log-log plot showing the dependence of contig length on the number of reads assembled into each contig.

A mixed sample of cDNAs obtained from ten tissues, including brain, gill, heart, kidney, liver, spleen, intestine, muscle, testis, and ovary, was prepared and sequenced using the 454 GS FLX titanium platform in one full PicoTiter plate. This sequencing run produced 749,954 raw reads with an average length of 235 bp (length range = 40 bp to 1,139 bp; Table 1; available at NCBI Short Read Archive, SRP020479). After adapters, short, and low-quality sequences were removed, we obtained 584,419 high-quality sequence reads with an average length of 206 bp. These results showed that 77.9% of the raw reads contained useful sequence data, which could be used for subsequent assembly. As a result, 86.4% of the clean reads ranged between 100 and 500 bp in length (Figure 1A).

**Table 1:** **Summary of 454 transcriptome sequencing and assembly for** ***C. semilaevis***

	Sequencing number	Bases (Mb)	Average length (bp)
Raw sequencing reads	749, 954	176.3	235.1
Clean reads	584, 419	120.5	206.2
Contigs	62, 632	17	272
Singletons	98, 262	17	173
Unigenes	150,039	32.5	216.3

The assembly of the trimmed and size-selected reads produced 62,632 contigs and 98,262 singletons. The length of the contigs ranged from 100 bp to 1,665 bp with an average length of 272 bp and an N50 of 303 bp (Figure 1B; Table 1). The average sequencing coverage, which was determined as the number of reads assembled into a particular contig, was 10.2. A positive relationship between the length of a specific contig and its coverage was observed in a randomly fragmented transcriptome (Figure 1C) [11]. Using cd-hit [13], we performed cluster analysis with a standard such that the sequences with a similarity to this transcriptome of >95% were clustered into one class; the longest sequence of each class was used as a representative sequence. A total of 150,039 representative sequences, which may be unigenes, were obtained.

The complete genome sequence of *C. semilaevis* has been obtained, in which a 477 M genome with a scaffold N50 size of 867 kb was assembled [1]. To assess our trancriptome assembly, we mapped the unigenes to *C. semilaevis* genome. Approximately 93.2% of unigenes exhibited significant hits on the genome. The mapping rates were 94.2% and 91.6% for isotigs and singletons, respectively. A high mapping rate indicated that our established assembly was assembly.

The result of assembly of our data was compared to other fish transcriptome by 454 pyrosequencing (Table 2) [10, 14, 15]. The average length of raw reads of the four fish transcriptomes was not as long as the desired length of the technique. This result suggested that the procedures used to construct genome libraries should be improved. Moreover, the average length of *C. semilaevis* contigs was shorter than that of the three other fishes. This result may be attributed to the following reasons. i) In sequence depth, *Oncorhynchus mykiss* and *Poecilia reticulate*, which sequenced more reads, *Oncorhynchus mykiss* and *Poecilia reticulate* produced longer contigs than *C. semilaevis*. ii) In the presence of alternative splicing regions, the assembly of long sequences may be impeded [16].

**Table 2:** **Compared with other fish transcriptomes using 454-pyrosequencing**

Species	Average length of raw reads	Numbers of raw reads	Total bases (M)	Average length of contigs	Number of contigs	Total bases (M)
*A. anguilla*	266	310, 079	82.5	530.6	19,631	10.4
*P. reticulata*	202.3	1, 665, 609	336.9	464.8	54,921	25.5
*O. mykiss*	344	1, 416, 404	447	662	151, 847	100.5
*Cv semilaevis*	235.1	749, 954	235.1	272	62, 632	17

### Annotation

Several complementary approaches were used to annotate the assembled sequences. The unigenes were initially compared with those in public protein databases by using BLASTX [17]. With this procedure, the gene names of 26,589 (17.7%) sequences were successfully assigned (Additional file 1). A low annotation rate was mainly attributed to a short unigene because the significance of the BLAST comparison partially depends on the length of query sequence; thus, short reads obtained from sequencing rarely match known genes [18]. In the present study, the annotation rate of short sequences (<300 bp, 14.5%) was lower than that of long sequences (>300 bp, 30.8%). Another reason accounted for low assignment percentage was the lack of information regarding flatfish. For example, only 349 sequences accounting for 1.31% of the total annotated sequences were annotated using the known information on flatfish. The three species with the most hits to Pleuronectiformes were *Paralichthys olivaceus* (129, 0.49%), *C. semilaevis* (97, 0.36%), and *Solea senegalensis* (57, 0.21%).

**Figure 2:**
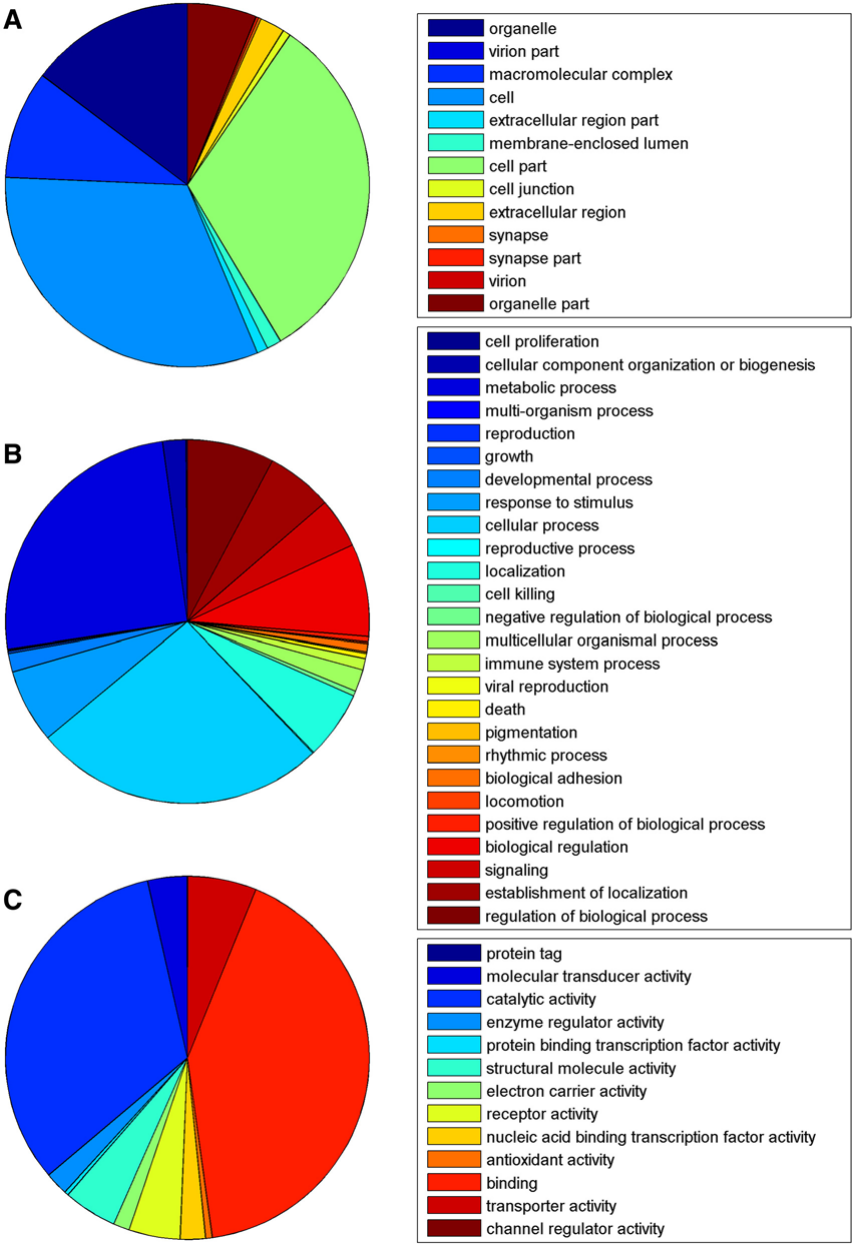
**Functional annotation of assembled sequences based on gene ontology (GO) categorization. (A)** Cellular component **(B)** Biological process **(C)** Molecular function.

The unigenes with matches in public protein databases were annotated with Gene Ontology (GO) annotation, which provides a dynamically controlled vocabulary and hierarchical relationships to represent information regarding molecular function, cellular component, and biological process [19]. Among the 26,589 unigenes, 3,451 were annotated with 17,113 GO terms (Additional file 2). We also found the following records: 1,921 annotated with a cellular component (GO ID: 0005575); 3,020 annotated with a molecular function (GO ID: 0003674); and 2,561 annotated with a biological process (GO ID: 0008150). In cellular component, genes involved in cell (GO ID: 0005623, 32%) and cell part (GO ID: 0044464, 32%) were the most abundant (Figure 2A). In molecular functions, binding (GO ID: 0005488, 42%) and catalytic activities (GO ID: 0003824, 33%) dominated the GO terms (Figure 2B). In biological process, the most abundant categories were cellular (GO ID: 0009987, 26%) and metabolic (GO ID: 0008152, 25%) processes (Figure 2C).

The Kyoto Encyclopedia of Genes and Genomes (KEGG) pathway approach used to perform high-order functional annotation was implemented using the web tool KASS [20]. A total of 2,362 unigenes were mapped to 186 different pathways (Additional file 3). Among these pathways, the three highest maps were metabolic pathways (KO01100), biosynthesis of secondary metabolites (KO01110), and microbial metabolism in diverse environments (KO01120).

### Functional genes involved in growth and immunity

As a valuable aquaculture fish, half-smooth tongue sole exhibits economical traits that are of great importance to aquaculturists. The sequences obtained by pyrosequencing and the annotation information provided significant data to determine economically important traits, including growth, reproduction, stress, and immunity.

The transcripts responsible for growth (GO: 0040007) were identified in our dataset. Seven sequences were identified as growth-related hormones. Among these seven sequences, three correspond to growth hormone-inducible transmembrane proteins, two correspond to growth hormone receptor 2, one corresponds to a predicted potassium channel subfamily K member, and the remaining sequence corresponds to a growth hormone-releasing hormone receptor.

MHC, a group of genes that encode for major histocompatibility antigens, functions in the immune response of vertebrates. Approximately 28 sequences have been annotated as MHC genes [21]. This finding is consistent with that of the variability of MHC genes.

Cytochrome P450 (CYP) is a part of a large family of heme enzymes that catalyze diverse chemical reactions, including epoxidation, hydroxylation, and heteroatom oxidation [22]. Steroidogenic enzyme is a member of the family P450 aromatase (P450arom); this enzyme is responsible for the conversion of androgen to estrogen and suppression of P450 arom gene expression; as a result, phenotypic sex reversal in fish occurs [23, 24]. The gender ratio of the cultivated half-smooth tongue sole was not 1:1 because the number of males was higher than that of the females. We identified 49 sequences annotated as P450; GO terms and KEGG pathways of these sequences were also detected.

### Transposable elements identification

**Figure 3:**
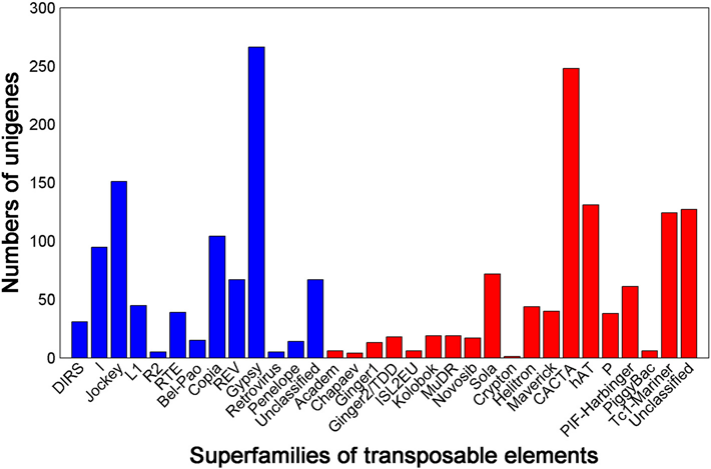
**Abundance distribution of transposable elements in the unigenes of** ***C. semilaevis*** . The blue bars represent retroelements while the red bars represent DNA transposons.

TEs can be divided into two general classes [25]: class I or retroelements are transposed *via* an RNA intermediate and class II or DNA transposons can directly manipulate DNA to propagate themselves into another site within a genome [26, 27]. A search on our transcriptome data revealed that 1,898 sequences contained putative TEs; among these TEs, 904 and 994 belonged to retroelements and DNA transposons, respectively (Figure 3, Additional file 4). The most common retroelements were Gypsy (266, 29.4%), Jockey (151, 16.7%), and Copia (104, 11.5%); the most common DNA transposons were CACTA (248, 24.9%), hAT (131, 13.2%), and Tc1-Mariner (124, 12.5%).

Previous studies reported that TEs are involved in the formation of the sex chromosomes of fish [28–30]. *C. semilaevis* contains a pair of well-differentiated sex chromosomes, and a large W chromosome is easily recognizable [31, 32]. However, the mechanism by which the W chromosome is formed in *C. semilaevis* remains unclear. In another study (unpublished), *C. semilaevis* contains a large number of TEs accumulated in the heterochromatic regions of the W chromosome. Our data sets are important to understand this phenomenon.

### SSR and SNP discovery

A total of 7,869 SSRs located in 6,997 sequences were identified in our 454 pyrosequencing data set. The most common repeat motifs were dinucleotides, which accounted for 64.3% of the total SSRs; other repeat motifs were trinucleotides (31.1%), tetranucleotides (3.5%), pentanucleotides (0.7%), and hexanucleotides (0.4%). On the basis of the distribution of SSR motifs, we found that AC motifs were the most common (20.2%); CAG and AAAC repeat motifs were the most abundant motifs in tri- and tetra-nucleotides, respectively.

Molecular markers should be developed to facilitate marker-assisted selection to optimize commercial species and improve the available genetic resources of species. Transcriptome sequencing is a rapid and effective method to identify SSR and SNP markers. These markers identified by transcriptome sequencing are possibly associated with specific traits. In our trancriptome sequencing, 7,869 SSRs were identified, but the effectiveness should be validated. Previous studies may provide references for validation. For instance, Gao *et al.* identified 4,952 putative SSRs in the transcriptome of blunt snout bream (*Megalobrama amblycephala*) by 454 pyrosequencing, and 116 of 160 (72.5%) SSRs have been validated by PCR. In addition, 71 (44.4%) of these SSRs were polymorphic across a panel of 40 individuals. The *C. semilaevis* transcriptomes in this study and our study showed that dinucleotide repeats accounted for the highest proportions (64.3% and 60.5%, respectively). However, the proportion of dinucleotide repeats is higher (5,291/6,501, 81.4%) in *Clupea harengus* transcriptome [33]; this result indicated that dinucleotide repeats are the most common SSR type.

PCR and sequencing errors resulted in false positives to detect SNP. We implemented strict criteria to reduce the effects of sequencing errors. These criteria were listed as follows: (1) Q value of the bases was restricted at <23; (2) minor allele frequency was >15%; and (3) the minimum number of minor allele reads was set at 2. Hence, a total of 21,234 putative SNPs and 13,370 putative single-nucleotide indels were identified. These putative SNPs included 14,333 transitions and 6,901 transversions. The overall frequency of all of the SNP types and indels in the transcriptome was 1 per 491 bp.

False positive should be considered when SNP is detected by high-throughput sequencing technology. The number of identified SNPs and the false-positive rate were directly affected by the criteria used to identify SNP. Numerous SNPs are usually obtained when low parameter settings are used, resulting in a low positive rate. In a previous study, 56,109 putative SNPs and 72,020 indels are detected in *M. amblycephala* [34] without considering the minor allele frequency; after a minimum minor allele frequency was set at >15%, the numbers decreased to 25,697 and 23,287, respectively. These results are similar to those in our study. We also examined the changes in the number of putative SNPs and indels when different numbers of minimal minor allele reads were applied. The number of putative SNPs and indels decreased, whereas the number of minor allele reads increased (Table 3). Moreover, the SNP false-positive rate decreased and the SNPs were more prevalent in the half-smooth tongue sole populations than in other species. Therefore, the SNPs with high numbers of supported reads should be selected when SNP marker development is performed.

**Table 3:** **The relationship of number putative SNPs and indels and number of minor allele reads**

Reads number	2	3	4	5	6	7	8
SNP number	21,234	8,284	4,631	3,013	2,076	1,535	1,222
Indel number	13,370	5,072	2,715	1,698	1,147	847	629

Reads number means the least reads number supporting the minor allele.

## Conclusions

We performed the *de novo* transcriptome sequencing of half-smooth tongue sole (*C. semilaevis*) by using a 454 FLX titanium platform. Our results revealed a large number of candidate genes potentially involved in growth, reproduction, and stress/immunity response. Putative TEs were detected and analyzed. Moreover, numerous SNPs and SSRs were identified and prepared to perform marker development. Our data set provided a useful resource for future genetic or genomic studies on this species.

## Methods

### Ethics statement

All of the experimental animal programs involved in this study were approved by the Ocean University of China’s Animal Care and Use Committee and performed in accordance with the experimental basic principles.

### Biological materials and RNA extraction

Six adult *C. semilaevis* (three males and three females) individuals were obtained from the Yellow Sea Aquatic Product Co., Ltd. and temporarily housed in the laboratory without feeding for 2 d. These individuals were euthanized using 300 mg/L tricaine methanesulfonate (MS222). Afterward, different tissues, including brain, gill, heart, liver, kidney, spleen, intestines (the end part), muscle, testis, and ovary, were removed, immediately frozen in liquid nitrogen, and stored at -80°C. Total RNA was extracted from these tissues by using Trizol reagent (Invitrogen, USA). The quantity and quality of the total RNA were determined by spectrophotometry (Bio-Spec-mini, Shimadzu, Japan) and gel electrophoresis. Equal quantities of high-quality RNA of each tissue from different individual fish were pooled for cDNA synthesis.

### cDNA library construction and pyrosequencing

cDNA library was constructed following the protocol described by Meyer *et al.* [8]. In brief, the RNAs from the same tissue of the six *C. semilaevis* individuals were pooled before the first-strand cDNA was synthesized using a SMART PCR cDNA synthesis kit (Clontech, USA). The second-strand cDNA was amplified using an Advantage® 2 PCR kit (Clontech, USA). The PCR products were purified using a QIA quick® PCR purification kit (QIAGEN, Germany).

cDNA samples were normalized using a TRIMMER cDNA normalization kit (Evrogen, Russia) to balance the number of transcripts with high and low expressions. After normalization was performed, cDNA samples were sheared by sonication using an ultrasonic crusher (JY-92IIDN, Ningbo Xinzhi Biotech, China) to produce fragments with a length ranging from 300 bp to 800 bp, which is the appropriate fragment size range in 454 pyrosequencing.

Oligonucleotide adaptors contain a barcode sequence to discriminate samples from different tissues. Before sequencing was performed, all of the libraries were combined into a single pool and 5 μg of the mixed cDNA sample was used in a full PicoTiter plate of 454 GS FLX titanium (Roche, Switzerland) according to the standard manufacturer’s instructions (Shanghai Oebiotech Co., Ltd., China).

### Sequence data analysis and assembly

The obtained raw reads were initially pre-processed by removing the adaptors and the primers using SeqClean (latest x86_64, Dana-Farber Cancer Institute) and Newbler (version 2.5.3, Roche). Low-quality reads were removed using Lucy (version 1.20p, -m50 –e 0.03 0.03 –w 30 0.03 10 0.1 –b 4 0.03) [35]. High-quality reads with a length of >50 bp were maintained. The trimmed and size-selected reads were then assembled using Newbler with the following parameters: “use duplicate reads”; “extend low-depth overlaps”; “minimum read length = 45 bp”; “read limited to one contig”; and “single ACE file”. After the assembly was constructed, contigs and singletons were subjected to cluster analysis using cd-hit (version 4.0) with the following parameters: -r 1 and –c 0.95. The longest sequence of each class was used as a representative sequence. The alignment between unigenes and *C. semilaevis* genome was performed using BLASTN with e-value < 1e–10.

### Sequence annotation

The unigenes were compared with the NCBI non-redundant protein database by using BLASTX. The alignment was processed using the following parameters: (1) e-value < 1e–5; (2) identity > 50%; and (3) number of matched amino acids >50. If the length of a unigene was <150 bp, the number of matched amino acids should be >70% of its own unigene. Gene function was assigned to each unigene based on the most common BLAST hit.

GO annotation was performed using Blast2GO, a software package that retrieves GO terms; this software package can be used to determine and compare gene functions [36–38]. KEGG pathways were assigned to the unigenes by using the online KEGG Automatic Annotation Server (KAAS; http://www.genome.jp/kegg/kaas/).

### Transposable elements identification

Putative TEs were identified based on a homology search. Our data sets were compared with RepBase 17.09 using tBLASTx with a threshold of 1e–5 [39]. The outputs were manually inspected, and significant matches to Simple Repeat, Pseudogene, and Integrated Virus were excluded.

### SSR and SNP discovery

SSRs were identified using the MISA program. The minimum number of repetitions of dinucleotide was set at six. By comparison, trinucleotide, tetranucleotide, pentanucleotide, and hexanucleotide were set at five. The maximum difference between two SSRs was 100 bp.

Putative SNPs and single-nucleotide indels were detected using a ssahaSNP program [40]. A putative SNP and indel site should satisfy the following conditions: (1) Q value of bases > 22; (2) minor allele frequency > 15%; and (3) minor allele was supported by at least two reads.

## Electronic supplementary material

Additional file 1: **Table BLASTTX annotation of assembled sequences.** The assembled sequences were compared against the public protein databases using BLASTX. (XLS 7 MB)

Additional file 2: **Table Gene Ontology annotations for** ***C. semilaevis*** . The sequences with matches with the public protein database were annotated with Geno Ontology annotation. (XLS 1 MB)

Additional file 3: **KEGG biochemical mapping for** ***C. semilaevis*** . The KEGG pathway approach was implemented using the web tool KASS. (XLS 78 KB)

Additional file 4: **Transposable elements identification for** ***C. semilaevis*** . Our data set were compared against RepBase using tBLASTx. (XLS 464 KB)
